# *Helicobacter pylori* Virulence Factors Exploiting Gastric Colonization and its Pathogenicity

**DOI:** 10.3390/toxins11110677

**Published:** 2019-11-19

**Authors:** Shamshul Ansari, Yoshio Yamaoka

**Affiliations:** 1 Department of Microbiology, Chitwan Medical College and Teaching Hospital, Bharatpur 44200, Chitwan, Nepal; shamshulansari483@yahoo.com; 2 Department of Environmental and Preventive Medicine, Oita University Faculty of Medicine, Idaigaoka, Hasama-machi, Yufu, Oita 879-5593, Japan; 3 Global Oita Medical Advanced Research Center for Health, Idaigaoka, Hasama-machi, Yufu, Oita 879-5593, Japan; 4 Department of Medicine, Gastroenterology and Hepatology Section, Baylor College of Medicine, 2002 Holcombe Blvd., Houston, TX 77030, USA; 5 Borneo Medical and Health Research Centre, Universiti Malaysia Sabah, Kota Kinabaru, Sabah 88400, Malaysia

**Keywords:** *Helicobacter pylori*, gastritis, peptic ulcer, gastric cancer, CagA, *cag*PAI

## Abstract

*Helicobacter pylori* colonizes the gastric epithelial cells of at least half of the world’s population, and it is the strongest risk factor for developing gastric complications like chronic gastritis, ulcer diseases, and gastric cancer. To successfully colonize and establish a persistent infection, the bacteria must overcome harsh gastric conditions. *H. pylori* has a well-developed mechanism by which it can survive in a very acidic niche. Despite bacterial factors, gastric environmental factors and host genetic constituents together play a co-operative role for gastric pathogenicity. The virulence factors include bacterial colonization factors BabA, SabA, OipA, and HopQ, and the virulence factors necessary for gastric pathogenicity include the effector proteins like CagA, VacA, HtrA, and the outer membrane vesicles. Bacterial factors are considered more important. Here, we summarize the recent information to better understand several bacterial virulence factors and their role in the pathogenic mechanism.

## 1. Background

*Helicobacter pylori*, one of the most common bacteria to colonize the gastric epithelium of about half of the world’s population, has been estimated to accompany humans for at least 100,000 years [1]. Based on epidemiological data, the bacterium has been categorized as a class-I carcinogen, because it is the strongest known risk factor for severe gastric complication development [2]. Geographic variation was found to influence infection prevalence between countries, and socio-economic status, urbanization level and poor sanitation during childhood has been linked to infection prevalence variation between countries [3]. A recent meta-analysis found that the estimated overall global prevalence was 44.3%, with the prevalence as high as 89.7% in Nigeria and as low as 8.9% in Yemen [4].

Although the exact route of bacterial transmission is unknown, documented evidence supports oral–oral or fecal–oral transmission from person to person between family members, and it shows a greater chance of acquiring the infection in the early years of life [5,6]. After its transition to the gastric lumen, *H. pylori* colonizes specific sites like the antrum and corpus. *H. pylori* has well-developed adaptation mechanisms to survive in the harsh gastric acid conditions and to establish a permanent infection (reviewed by Ansari and Yamaoka [7]).

Once the permanent infection is established in the stomach, several gastro-duodenal complications like chronic gastritis, peptic ulcer diseases, gastric cancer, and gastric mucosa-associated lymphoid tissue (MALT) lymphoma may develop [8]. However, the frequency of patients developing severe complications is very low; it has been estimated that less than 1, 10–300, and 100–1000 patients develop MALT lymphoma, gastric cancer, and peptic ulcer diseases, respectively, among every 10,000 patients infected with *H. pylori* [9]. It has been found that approximately 70% of all gastric ulcers and up to 80% of all duodenal ulcers are caused by *H. pylori* infection, which is a significant factor causing non-iatrogenic peptic ulcer diseases. The risk of peptic ulcer development increases with previous history of *H. pylori* infection even after its successful eradication compared with non-infected individuals [9]. However, investigations indicate that the recurrence of peptic ulcer diseases decreases with the successful eradication of *H. pylori* infection compared to non-cured patients [10]. The results of a study on the relationship between ulcer disease recurrences and *H. pylori* eradication status found that the recurrence rate of gastric ulcers and peptic ulcers were 4% and 6%, respectively, in successfully cured patients compared to 59% and 67%, respectively, in non-cured patients [10]. However, the development of gastric complications like peptic ulcer diseases and gastric cancer is a long-term process that may take several decades, and it is a multifactorial process influenced by gastric environmental, host genetic, and bacterial virulence factors [11].

## 2. Virulence Factors Associated with Escape to High Acidic Environment

After transit to the gastric lumen, the *H. pylori* encounters extremely harsh conditions of pH around 2.0. However, *H. pylori* possesses several factors like urease, bacterial shape and flagella mediating motility to interact with the harsh gastric environment (Table 1). The acidic conditions help the bacteria to express some genetic determinants that neutralize the acidic environment (reviewed by Ansari and Yamaoka [7]).

### 2.1. Urease

A large amount of intracellular urease is produced by *H. pylori*, constituting around 10% of the total bacterial protein production. In addition to intracellular urease, *H. pylori* also contains extracellular urease on the bacterial surface due to the lysis of some bacteria in the stomach [12,13].

Urease-catalyzed urea hydrolysis (endogenous and exogenous) results in ammonia (NH_3_) and carbamate production, which is spontaneously decomposed to yield another ammonia (NH_3_) and carbonic acid (H_2_CO_3_). The carbonic acid is broken down to CO_2_ and water (H_2_O) molecules. Ammonia in its protonated form (NH_4_+) neutralizes stomach acidity and plays an important role in providing a favorable nearly neutral micro-environment around *H. pylori* [14]. CO_2_ is converted to bicarbonate (HCO_3_^−^) and H^+^ in the periplasmic space by periplasmic α-carbonic anhydrase, maintaining the periplasmic pH close to 6.1 via an acid acclimation mechanism. In this way, NH_3_ and CO_2_ production provides the necessary environment for *H. pylori*’s gastric survival [15,16]. However, continuous urease expression is required by bacteria for successful colonization even after its survival adaptation in the stomach [17].

In addition to its role in acid neutralization, several in vitro studies uncovered urease’s role and its catalytic products in pathogenicity establishment. Ammonia production disrupts the tight cell junctions, breaches the cellular integrity, and damages the gastric epithelium [18,19]. CO_2_ protects the bacterium from the bactericidal activity of metabolic products like nitric oxide and intracellular killing by phagocytes [20]. Urease was recently reported to possibly contribute to tumor growth and metastatic dissemination by inducing angiogenesis, or new blood vessel formation from pre-existing vasculature, and playing a key role in gastric cancer progression [21]. Chronic *H. pylori* infection has been found to induce hypoxia-induced factor (HIF), which contributes to the development and progression of several cancers. A recent study showed that the *H. pylori* urease activated the PI3K-AKT-mTOR pathway in gastric cells. The activation of this pathway increases HIF-α expression [22]. Moreover, *H. pylori* urease was found to drive the differentiation of endothelial cells by producing reactive oxygen species and activating the lipoxygenase pathway via pro-inflammatory properties, contributing to *H. pylori* infection progression to gastric carcinogenesis [23]. Furthermore, *H. pylori* urease was shown to bind to major histocompatibility complex (MHC) class II molecules and induce cell apoptosis [24].

### 2.2. Bacterial Shape

A study of the bacterial shape’s role in movement showed that a mutation in the cell shape determinants causing the bacteria to adopt a straight rod morphology reduced the speed of bacterial movement by 7–21% [25]. Moreover, the results of another study using a mouse infection model showed that the mutant curved *H. pylori* were outcompeted by wild type helical *H. pylori* [26]. These studies suggest that the helical shape is important for the bacterium to penetrate into and move within the viscous mucous layer and for *H. pylori*’s efficient colonization.

The characteristic helical shape of bacteria which provides the bacterial motility also helps to cope with the harsh acidic conditions and adapt to different gastric environments [27]. After transiting to the stomach, the bacteria move from acidic conditions to the mucus layer, which provides a protective layer, before finally moving to the gastric epithelium to establish colonization and infection [25]. The helical bacterial shape facilitates the rapid corkscrew-like bacterial movement within the less acidic mucus layer, allowing the bacterium to escape the extremely low gastric pH [28]. Moreover, another study highlights the importance of the bacterial helical shape and motility for penetrating the mucus layer and colonizing the gastric epithelium [29].

### 2.3. Flagella Mediating Motility

The flagellum is the locomotory organ that enables the bacterium to move in the ecological niche. *H. pylori* normally possesses two to six sheathed flagella about 3 μm long at one pole [30]. Despite providing harsh conditions, the acid exposure in the gastric niche also activates some proteins that help to escape from the danger. It has been shown that acid exposure activates flagellin, the flagellar proteins leading to enhanced motility. In a study by Merrell et al., the larger percentage of acid-exposed bacteria displayed significantly higher speeds compared to non-acid-exposed bacteria [31].

The number of flagella plays a crucial role in the bacterial cell speed; bacteria with more flagella can move faster compared to cells with fewer flagella. One study demonstrated a 19% increase in bacterial speed in bacteria possessing four flagella in a viscous environment compared to cells with three flagella [25]. The bacterial cells with greater motility may be deposited with higher density on the gastric epithelium, triggering a higher inflammatory response. It has been found that strains with greater motility increase the sialic acid-binding adhesion (SabA)-mediated interaction of *H. pylori*, providing a synergistic effect for pathological outcome severity in patients [32].

After infection, the toll-like receptor (TLR) 5 recognizes the flagellin composed of D0, D1, D2, and D3 domains. TLR5 recognition is important for the immunological events necessary for the inflammatory process. A recent study by Forstneric et al. demonstrated the role of flagellin in the TLR5 recognition evasion. In the study, the residues within the domain D0 play a crucial role in flagellin’s escape from TLR5 recognition [33].

Therefore, urease, bacterial shape, the number of flagella, and motility allow the bacteria to escape the harsh gastric conditions and help establish persistent infections.

**Table 1:** Virulence factors necessary for *H. pylori* mediated pathogenicity.

Virulence Factors	Mechanism	Effects	References
**Acid escape virulence factors**			
Urease	Production of NH_3_ and CO_2_	Neutralizes the gastric acidity	[14,15,16]
		NH_3_ damages the gastric epithelium	[18,19]
		CO_2_ protects the bacteria from killing by metabolic products	[20]
	Angiogenesis	Causes gastric cancer progression	[21]
	Activation of PI3K-AKT-mTOR pathway	Enhances the progression of cancers	[22]
Bacterial shape	Helical bacterial shape	Enhances the bacterial penetration into the mucous layer protecting the bacteria	[28]
Flagella	Motility	Helps bacteria to move away from the acidic environment	[30]
	Flagellin activation	Displays higher motility that protects the bacteria	[31]
**Epithelial cells colonizing factors**			
BabA	Binds with the epithelial cell receptor Leb	Mediates bacterial attachment and colonization	[34]
		Enhances CagA translocation	[35]
		Induces double strand breaks in the host cells	[36]
SabA	Binds with sialyl-Lex antigen	Mediates bacterial attachment and colonization	[37]
OipA	Bacterial adherence to the gastric epithelium	Damages mucosal layer	[38]
		Induces interleukin (IL)-8 expression	[38]
		Causes host cell apoptosis	[39]
HopQ	Bacterial adherence to the gastric epithelium	Inhibits immune cell activities	[40]
**Epithelial cells pathogenicity factors**			
*cag*PAI	Encodes syringe like T4SS	Translocation of CagA and peptidoglycan	[41,42,43,44]
CagT	Acts as core complex protein in T4SS	Helps in the translocation of CagA	[45]
CagY	Binds with integrin	Modulates the immune response to promote the bacterial persistence	[46]
		Alters T4SS functions	[47]
Cagζ	Unknown	Associates with T4SS function and mediates CagA delivery	[48]
CagL	Acts as core complex protein in T4SS and binds with integrin	Helps in the translocation of CagA	[42,49]
		Induces IL-8 expression	[50]
CagA	Phosphorylation of tyrosine	Causes cellular proliferation	[51]
		Causes IL-8 expression	[52]
		Causes cell elongation	[53]
		Down regulates the heat shock protein 1	[54,55]
VacA	Vacuolization of epithelial cells	Causes cell vacuolization	[56]
		Causes cell necrosis	[57]
		Causes cellular apoptosis	[58,59,60]
	Endoplasmic reticulum stress	Enhances activation of autophagy and increased cellular death	[61]
HtrA	Acts as protease	Degrades mis-folded proteins	[62,63]
		Enables delivery of CagA	[64,65,66,67,68]
		Cleaves the tight junction proteins (occluding, claudin-8, and E-cadherin)	[64,65,66,67,68,69,70,71]
Outer membrane vesicles	Clathrin dependent and independent internalization	Protects pathogen from the toxic effects of reactive oxygen species	[72]
		Impairs cellular functions	[73]
		Induces dendritic cell functions	[74,75]
γ-glutamyl transpeptidase	Transpeptidation and amino acid synthesis	Enhances cell apoptosis	[76]
		Inhibits cellular proliferation	[77]
		Arrests cell cycle	[78]
	VacA dependent vacuolation of epithelial cells	Epithelial cell destruction	[79]

## 3. Virulence Factors Associated with Colonization of Epithelial Surfaces

The outer membrane proteins like blood group antigen-binding adhesin (BabA), SabA, outer inflammatory protein (OipA), *H. pylori* outer membrane protein (HopQ), and other proteins interact with the receptors found on the host epithelial cells, playing a key role in pathological events of persistent infections (Table 1). This interaction also provides several benefits to infecting *H. pylori* strains. The interaction protects the bacterium from washing out during mucus shedding, provides nutrient access to the bacteria, and promotes delivery of bacterial toxins and other effector molecules to the host cells [80,81,82].

### 3.1. Blood Group Antigen-Binding Adhesin

BabA, an outer membrane adhesin protein, plays a crucial role in bacterial attachment, and it makes a significant contribution to cellular pathogenicity (reviewed by Ansari and Yamaoka [34]). BabA is an important protein of outer membrane protein (OMP) family with an approximate molecular weight of around 75–80 kDa. Two closely related paralogs, BabB and BabC have been identified [83,84].

*H. pylori* adheres to the epithelium with the help of BabA as an adhesin on the bacterial surface that interacts with di-fucosylated glycan found on Leb and mono-fucosylated glycan found on H1-antigen, A-antigen, and B-antigen of blood groups O, A, and B respectively [85,86,87]. Although the BabA-mediated attachment provides survival adaptation and persistent infections, the functions of BabB and BabC are not yet known. The *bab* gene sequence analysis revealed that there is variability in the middle sequence region and extensive 5′ and 3′ region conservation, particularly between *babA* and *babB*. The sequence variability in the middle region of *babA* suggests that this is the location of BabA’s receptor-binding function [84,88]. The 5′ and 3′ conservation in *bab* gene sequences undergo RecA-dependent intragenomic recombination between these homologous genes (*babA*, *babB* and *babC*), resulting in chimeric genes that may provide benefits to the organisms. The formation of the chimeric gene *babB/A* has been shown to convert non-Leb binding strains to Leb binding strains, or it can abolish *babA*-dependent adhesion [89,90].

The BabA binding with Leb has been shown to enhance CagA translocation [35] and induce VacA, γ-glutamyl transpeptidase, and cag-pathogenicity island (*cag*PAI)-independent double-strand breaks in the host cells [36]. In Western countries, infection with BabA producing strains has been associated with an increased risk for peptic ulcer disease development [91,92]. A recent study highlighted the adherence of strains with a high avidity mediated by BabA-positive pediatric ulcerogenic *H. pylori* strains [93].

Based on the binding preferences with ABO blood group antigens, the BabA-positive *H. pylori* strains can be of two types. The “specialists” prefer to bind only blood group O-specific glycans, while the “generalists” bind blood group O and blood group A and B-specific glycans [87]. The capability of both specialist and generalist strains to bind with blood group O-specific glycans explains why people with blood group O are at a high risk for developing duodenal ulcer diseases [87].

### 3.2. Sialic Acid-Binding Adhesin

The OMP SabA binds to gangliosides with fucose substitutions of the N-acetyllactosamine like the dimeric sialyl-Lex antigen. SabA also binds to N-acetyllactosamine-based antigens with terminal α3-linked Neu5Ac, with preferential binding to gangliosides with long N-acetyllactosamine chains [37]. Recently, minor SabA binding gangliosides of the human gastric mucosa, Neu5Aca3-neolactohexaosylceramide and Neu5Aca3-neolactooctaosylceramide, have been identified. They may provide new insight into the molecular basis for the *H. pylori* chronic infection in human hosts [94].

An enhanced *H. pylori* colonization density in Leb-negative individuals was reported based on interactions between SabA adhesin and sialylated gastric glycoconjugates [95]. The *sabA* gene sequence can undergo homologous recombination with the homologous gene called *sabB*, and it can also happen to some extent with *hopQ* [96].

Several studies reported an association between *sabA* expression and an increased risk of chronic gastritis [97,98,99], intestinal metaplasia, corpus atrophy and even gastric cancer [91,100]. *H. pylori*-mediated gastric inflammation was shown to alter the gastric mucosa glycosylation with an upregulation of sialyl-Lex antigens and promoting the SabA mediated bacterial attachment to the gastric mucosa [100,101]. Therefore, increased expression of high-affinity SabA binding glycoconjugates in inflamed gastric epithelium supports enhanced adherence of SabA-positive *H. pylori*, allowing bacterial persistence and gastric pathogenicity establishment [100]. The results of a study conducted by Mahadavi et al. [100] suggest a close relationship between the presence of *sabA*, the cag-pathogenicity island, and *babA*. According to some authors, the combination of SabA with other virulence factors like OipA and BabA gives the best predictive model by which gastric cancer patients can be distinguished from duodenal ulcer patients and normal individuals [102].

### 3.3. Outer Inflammatory Protein (OipA)

OipA is another virulence protein belonging to the *H. pylori* OMP family, and it is encoded by the *oipA* gene. The number of CT dinucleotide repeats in the 5′ signal peptide regulates the expression of OipA via a slipped strand mispairing system. The status is designated as “on” when a functional protein is expressed, while expression of a non-functional protein is designated as “off” [103].

The strains expressing a functional OipA are reportedly involved in bacterial adherence to the gastric epithelial cells with mucosal damage [38] and association with host cell apoptosis [39], interleukin (IL)-8 induction [38], duodenal ulcers [104], and gastric cancer development [105,106]. A study report highlighted the *oipA* gene’s association with chronic gastritis development. Strains expressing functional OipA were most frequently isolated from patients with gastric cancer in Western countries [107].

Furthermore, the results of another study reported that patients infected with strains with “on” status *oipA* possess a higher risk for peptic ulcers and gastric cancer development rather than developing gastritis or functional dyspepsia [108]. A recent study showed that enhanced adherence to epithelial cells and the translocation of CagA in the presence of functional *cag*PAI is exhibited by the OipA positive strains [104]. The existence of *oipA* together with *cagA* have been suggested to contribute to proliferation and damage to the normal cellular integrity associated with β-catenin signaling [109,110]. Moreover, *H. pylori* OipA was reported to reduce IL-10 expression and dendritic cell (DC) maturation, contributing to chronic *H. pylori* infection establishment [111].

### 3.4. Helicobacter Pylori Outer Membrane Protein (HopQ)

HopQ is another *H. pylori* OMP. Sequence analysis of *hopQ* from unrelated strains revealed two genotypes classified as type I and type II, which exhibit a high level of genetic diversity [112,113]. *H. pylori* HopQ contributes to bacterial adherence to epithelial cells interacting with various members of the carcinoembryonic antigen-related cell adhesion molecules (CEACAM) family expressed on the gastric epithelium specifically during gastritis and gastric cancer development [114,115,116].

In 2005, Cao et al. reported that the *H. pylori* HopQ type I genotype is associated with increased peptic ulcer development in the US population [117]. Consequently, another study also found a significant association of HopQ type I in gastric ulcers [118] and in gastric cancer development [118,119]. Similarly, studies also found an association between HopQ type II and gastric cancer development [120].

The recent studies highlighted that the interaction between HopQ and CEACAM contributes to gastric colonization [114] and facilitates translocation of the CagA protein into the gastric epithelium to induce pathogenicity [114,116,121,122]. A recent study showed that the interaction between HopQ and CEACAM plays a significant role in inhibiting immune cell activities [40]. Moreover, the association of multiple types of *H. pylori* HopQ (type I and II) has been recently found in the development of B-cell non-Hodgkin lymphoma and HopQ type II with a higher prevalence [123].

Therefore, OMP adhesins provide bacterial attachment to host cells and provide an additional role in pathogenicity.

## 4. Virulence Factors Associated with Gastric Epithelial Cell Pathogenicity

### 4.1. Cag-Pathogenicity Island (cagPAI)

*H. pylori cag*PAI is an approximately 40 kb long chromosomal region that contains up to 32 genes encoding a multicomponent bacterial type IV secretion system (T4SS) and an effector protein like CagA [41,124,125]. The T4SS forms a syringe-like structure that makes contact with the host epithelium and translocates the effector protein CagA and peptidoglycan into the epithelium [41,42,43,44]. Electron microscopy revealed T4SS to be a core structure of 41 nm protruding from the bacterial surface [49,126]. At least 17 genes must be expressed to encode the intact T4SS for its essential functions, and 14 genes must be expressed to fully induce IL-8 secretion [127,128]. The strains expressing the intact *cag*PAI are associated with the development of several disorders like chronic gastritis, peptic ulcer diseases, and gastric cancer [129,130,131,132,133]. Moreover, a recent study found that the strains recovered from the ulcer patients contained a significantly higher prevalence of all individual genes and intact *cag*PAI compared to strains recovered from non-ulcer patients [134].

Some of the *cag*PAI components are essential for T4SS function or they are important for CagA translocation (Table 1). CagT is essential for translocation of the effector protein CagA into the epithelial cells [45], while others like CagY act as an immune-sensitive molecular regulator that modulates the immune response to promote bacterial persistence and alter T4SS function [46,47]. Cagζ (Cag1), a membrane protein in T4SS, is closely associated with T4SS function, IL-8 expression induction, and CagA delivery to host cells [48]. A recent study uncovered CagQ’s role as a membrane protein in T4SS for maintaining CagA expression and CagA-induced apoptotic effects [135].

Another T4SS component, CagL, a pilin-like component encoded by the *cagL* gene (HP0539), is expressed on the surface of *H. pylori* in a T4SS-dependent manner [124,125] and in collaboration with the human integrin β1-containing receptors. In particular, integrin α5β1 is necessary for the CagA translocation [42,49]. The tripeptide motif arginine-glycine-aspartic acid (RGD) of CagL at residues 76–78 together with the RGD helper sequence, which is a neighboring surface-exposed FEANE (phenylalanine-glutamic acid-alanine-asparagine-glutamic acid) motif was proposed to be essential for the interaction of T4SS with integrin receptors for CagA translocation into host cells [136]. This RGD-dependent binding of CagL to integrins was also shown to trigger intracellular signaling pathways inducing cell pro-inflammatory responses that are CagA translocation-independent [46,137,138]. Recent studies found an association of novel CagL variants with chronic gastritis and peptic ulcer disease (PUD) development [139,140]. Particular polymorphisms upstream of the RGD motif at amino acid residues 58–62, called the CagL hypervariable motif (CagLHM), are correlated with severe disease progression in a geographically-dependent manner [141,142]. Therefore, *cagL* deletion was shown to phenocopy complete *cag*PAI knockout in many respects because of its role as an essential T4SS component [43,143]. This behavior was suggested due to the strain being unable to form a functioning T4SS in response to host cell contact without CagL [49,144]. Therefore, in addition to CagA, the strain will not be able to translocate the peptidoglycan, DNA, or precursors of lipopolysaccharide into host cells [145,146,147,148]. The results of a recent study demonstrated that in addition to playing a crucial role in the translocation of CagA, the *H. pylori* CagL may also be responsible for *H. pylori*-induced IL-8 expression via the transforming growth factor (TGF)-α activated epidermal growth factor-receptor (EGF-R) signaling pathway, *H. pylori*-induced hummingbird phenomena (elongation of the cells), and the bridging of the T4SS to its human target cells [50].

The effector protein CagA is 125–145 kDa. It is expressed by the highly virulent *H. pylori* strains possessing *cag*PAI, and it is absent from less virulent *cag*PAI negative strains. The synthesized CagA is translocated into the gastric epithelial cells via T4SS. Although *H. pylori* synthesizes an abundant amount of CagA, a relatively low amount of it is translocated inside the host epithelial cells [149]. The translocated intracellular CagA levels are also regulated by autophagy and the ubiquitin-proteasome system, which degrades the translocated CagA [150,151]. The biological activity of CagA is determined by the types and number of well-characterized EPIYA (glutamic acid-proline-isoleucine-tyrosine-alanine) sequences containing EPIYA-motifs at the C-terminal region. *H. pylori* isolates recovered from Western countries usually possess CagA with EPIYA-A, -B, and -C where the EPIYA-C region can be up to three units long, while the CagA in *H. pylori* strains recovered from the East Asian countries possess EPIYA-A, -B, and -D sequences. The first two EPIYA sequences, EPIYA-A and EPIYA-B, are carried by almost all CagA, and the third EPIYA sequence (either EPIYA-C in Western strains or EPIYA-D in East Asian strains) is associated with geographic, genotypic, and virulence characteristics [152]. After its translocation into the host epithelial cells, the EPIYA-motifs of CagA undergo tyrosine (Y)-phosphorylation via various cellular kinases like Csk, c-Src, and c-Abl [51,153]. The phosphorylated tyrosine interacts with the Src homology 2 phosphatase (SHP2) or the adapter protein Grb2 [52,154,155] and hinders cell–cell adhesion, cellular proliferation, IL-8 expression, and cellular elongation via the activation of various cell signaling pathways like Ras-ERK MAP kinases (Rap1 → B-Raf → Erk) and Wnt-β-signaling [51,52,53]. A recent study by Li et al. demonstrated that CagA stimulates YAP signaling pathway activation leading to gastric tumorigenesis in AGS cells. This in vitro result was also supported by the finding that *H. pylori* infection could enhance YAP expression activation in concert with E-cadherin suppression in chronic gastritis tissues infected with *H. pylori* compared to *H. pylori* negative patients [156].

The CagA with EPIYA-D motif has stronger binding affinity with SHP2 than the EPIYA-C motif [157]. Therefore, the strains containing CagA with EPIYA-A, -B, and -D motifs are considered more virulent than strains containing CagA with EPIYA-A, -B, and -C motifs. In a recent meta-analysis, CagA with a single EPIYA-D motif was significantly associated with a 1.91 fold increased gastric cancer risk in Asia compared with one EPIYA-C motif, and the CagA with two or more EPIYA-C motifs (EPIYA-A, -B, -C, -C or EPIYA-A, -B, -C, -C, -C) was associated with a significantly higher risk for PUDs in Asian countries and gastric cancer (OR (odd ratio) = 3.28) in the US and Europe [158]. In addition to the type of EPIYA-motifs, strains with amino acid polymorphism within the Western-specific EPIYA-B motif like EPIYT-B may influence CagA activity, reducing the ability to induce hummingbird phenomena and IL-8 expression conferring lower risk for gastric cancer and a higher risk for duodenal ulcer development [159]. A recent study demonstrated that secreted H. pylori CagA can induce caudal type homeobox 1 (CDX1) expression, which is a homeobox transcription factor that plays an important role in human intestine development and maintenance [160,161]. CDX1 activation promotes cell proliferation, invasion/migration, intestinalization of gastric epithelial cells, and stem cell-like phenotype induction leading to cancer development and failure of common gastric cancer chemotherapies [160]. Recently, another study discovered CagA-mediated downregulation of heat shock protein 1 (HSP1) expression after H. pylori infection [54]. CagA-dependent acute HSP1 suppression represses the host immune response, so H. pylori may escape from the immune response and enhance infection establishment [55]. Although cagPAI positive H. pylori is considered as the strongest risk factor for the development of the gastroduodenal complications, the precise mechanism behind the development of severe complications is still not completely understood. Recently, a study has pointed out the role of H. pylori cagPAI in the expression of Lrig1 (leucine-rich repeats and Ig-like domains 1) in a CagA and CagE-dependent manner. Lrig1 is a transmembrane protein that acts as an intestinal stem/progenitor cell marker. The study demonstrated a significant increase in Lrig1-positive cells in the premalignant lesions (atrophic gastritis and intestinal metaplasia) in the antrum and corpus compared with that in the normal mucosa and stomach lining, which indicates the possible contribution of these cells to the ability of H. pylori to cause injury and promote carcinogenesis in the stomach [162].

### 4.2. Vacuolating Cytotoxin (VacA)

VacA is an important *H. pylori* pore-forming cytotoxin that plays a crucial role in pathogenicity by interacting with gastric epithelial cells [163,164]. The ability of this toxin to induce vacuole formation in eukaryotic cells led to it being named VacA. Initially, VacA is formed as a 140 kDa pro-toxin that is secreted through the auto-transporter pathway. The mature 88 kDa secreted toxin undergoes limited proteolysis to yield two fragments: p33 and p55 [165].

The three heterogenic regions of VacA have significant sequence variation. The “s” region represents the sequence variation in the amino-terminal signal sequence. The “m” region represents the amino acid variation located in the middle of the p55 domain. The “i” region was recently identified in a survey conducted in the Iranian population, and it represents the amino acid variation in the intermediate region located between the “s“ and “m“ region in the p33 domain [166,167,168]. The sequence variation of VacA isolated from the clinical *H. pylori* isolates indicates the s1a, s1b, s1c, and s2 sub-families of the “s“ region. m1 and m2 are sub-families of the “m“ region. i1, i2, and i3 are sub-families of the “i“ region [166,168,169,170]. The allelic diversity was reviewed in detail by Tran et al. [171].

The studies have revealed that the combination of different sequences in the three regions can determine the capability of vacuolation. Moreover, the genotype possessing the combination s1/m1 exhibits high vacuolating activity, and the genotype s1/m2 has intermediate activity. On the other hand, no vacuolating activity was shown by the genotype s2/m2 [166]. This finding indicates that the vacuolating capability of VacA with the s2 genotype is lower than the s1 genotype. The s2 region contains an additional 12 amino acids in the N-terminal sequence causing the predominant hydrophilic nature compared to the strongly hydrophobic s1 region, which most probably causes impairment in the anion-selective channel formation and cell vacuolation [170]. The lower vacuolating activity was also supported by the fact that VacA export with the s2 genotype from the cytoplasmic membrane to the periplasmic space is less efficient compared to s1 genotypes [172]. The strains with the s1 sequence were reported to secrete higher amounts of VacA that may be caused by elevated *vacA* transcription than s2 type strains [173]. Therefore, these differences could cause the higher activity of vacuolation by strains possessing VacA with the s1/m1 genotype.

In clinical *H. pylori* isolates from ulcer patients, a higher prevalence of VacA with s1a, m1, and i1 genotypes have been detected compared to patients with non-ulcer diseases [134]. A meta-analysis conducted in Western populations found that the individuals pose an increased risk for gastric cancer development if they are infected with *H. pylori* harboring *vacA* with s1 or m1 regions [174]. On the other hand, in Middle Asia and Middle East Asia, patients infected with *vacA* i1 type harboring *H. pylori* are associated with a high risk for gastric cancer development (OR = 10.9–15.0) [175]. The antibody against VacA is associated with an increased risk for gastric cancer development [176]. A recent meta-analysis conducted by Li et al. observed an association of a VacA antibody with peptic ulcer disease and gastric cancer risk, which suggests the role of VacA as a biomarker for the prediction of peptic ulcer disease and gastric cancer risk [177]. Furthermore, a strong antibody response to *H. pylori* VacA is significantly associated with the risk of extra-gastric diseases such as colorectal cancer development, particularly in African Americans [178].

VacA plays several roles in cellular pathogenicity (Table 1), and it is considered a multifunctional toxin eliciting multiple effects on host cells like vacuolization and cell necrosis [56,57]. Numerous studies have also shown cell apoptosis induction by VagA through the mitochondrial pathway in gastric epithelial cells [58,59,60]. An additional apoptotic potential was elaborated in a study, which provided novel evidence that VacA triggers the endoplasmic reticulum stress response to activate autophagy and increased cellular death of AGS cells [61]. The studies have found that the 148 amino acid segment localized at the middle region exploits the cell binding specificity of VacA, and strains harboring VacA have the best survival for *H. pylori* inside the gastric epithelial cells [179,180,181]. A recent study reported that the VacA promotes bacterial survival independent of CagA accumulation [151]. This survival efficacy was determined in a recent study where the *H. pylori* VacA can play an important role in a transient receptor potential membrane channel mucolopin 1 (TRPML1) activity that inhibits the lysosomal and autophagic killing of bacterial cells to promote the establishment of an intracellular niche that allows for bacterial survival [182].

### 4.3. High-Temperature Requirement Protein (HtrA)

Organisms are continuously exposed to oxidative and heat stress that can kill them. However, organisms tolerate these stresses and degrade intracellular irreversibly misfolded proteins that may be toxic to the organisms [62,63]. The protease HtrA plays an important role in neutralizing the effects of these stress responses in both prokaryotes and eukaryotes like humans [183,184,185,186]. When encountering denatured or misfolded proteins, HtrA undergoes oligomerization and conformational changes to switch to proteolytic activity (Table 1). The HtrA protein is generally transported in the periplasmic space, where they form proteolytically active oligomers which exhibit both protein quality control and chaperone functions [187,188]. However, the HtrA protein is transported extracellularly in *H. pylori* [62,189,190]. *H. pylori* HtrA is highly resistant to temperature and pH variations, which indicates its ability for adaptation and causing colonization and persistent infection even in harsh stomach conditions [191,192].

The extracellularly exported HtrA enables the bacteria to deliver the virulence factor like CagA to the basolateral membrane of host cells despite infection initiation at the apical side [64,65,66,67,68]. The serine protease activity of the *H. pylori* HtrA protein was found to cleave the proteins of tight junctions like occludin, claudin-8, and the E-cadherin molecules on the gastric epithelial cells, which are important adherens junction proteins and tumor suppressors. Moreover, their destruction has been strongly connected with the progression and metastasis of gastric tumors in humans [64,65,66,67,68,69,70,71]. The findings have also suggested that the HtrA-mediated E-cadherin destruction causes the disruption of the epithelial barrier and transmigration of *H. pylori* across the polarized gastric epithelial linings [65,70,193]. The results of a recent study found that the htrA gene in *H. pylori* is one of the highly conserved genes to be found in isolates from Europe, Asia, North America, and South America. This conservation suggests that there is little or no effect on the htrA gene region over the course of *H. pylori* and human co-evolution like the *H. pylori* MLST housekeeping genes [68].

### 4.4. Outer Membrane Vesicles

Outer membrane vesicles (OMVs) are characterized as blebs of 20–300 nm in diameter which are naturally secreted by several Gram-negative bacteria including *H. pylori* during their growth in logarithmic phase [194,195,196]. Since OMVs are released from the outer membrane of bacteria, they contain several outer membrane specific phospholipids like phosphatidylglycerol (PG), phosphatidylethanolamine (PE), lyso-PE (LPE), phosphatidylcholine (PC), lyso-PC (LPC), cardiolipin, lipopolysaccharides, and several bacterial virulence factors like adhesins, proteases, and toxins [197,198,199,200,201]. After their release, these OMVs are taken up by gastric epithelial cells via clathrin-dependent endocytosis or a clathrin-independent mechanism (lipid raft-mediated mechanism) [202]. Moreover, the *H. pylori* OMVs have been reported to protect the pathogen from the toxic effect of the reactive oxygen species respiratory burst [72].

OMVs have been detected intracellularly and extracellularly in gastric biopsy specimens [199,200,203]. In addition to protecting the pathogen, they have been suggested to promote infection, impair cellular function, and modulate host immune defenses via immunosuppressive cytokine IL-10 production by human peripheral blood mononuclear cells and via apoptosis in Jurkat T cells [73,204,205]. Similarly, *H. pylori* OMVs have been also found to induce dendritic cells by upregulating the expression of co-stimulatory molecules and expression of more heme oxygenase-1 via the activation of Akt-Nrf2 and mTOR-κB Kinase–NF-κB pathways [74,75]. Moreover, the studies have detected *H. pylori* OMVs in the gastric juice of patients with gastric cancer [206,207]. In a recent study conducted by Choi et al., the gastric juice collected from gastric cancer patients showed a significantly higher amount of both *H. pylori* cells and the *H. pylori*-derived OMVs compared to healthy controls. Furthermore, these *H. pylori*-derived OMVs also induced inflammation in the mouse model, inducing gastric cancer development [207].

### 4.5. H. pylori γ-Glutamyl Transpeptidase

*H. pylori* γ-glutamyl transpeptidase (gGT) is an enzyme that catalyzes the transpeptidation and hydrolysis of the γ-glutamyl moiety of glutathione and glutathione-conjugated compounds to amino acids [208]. Although gGT is an essential component for *H. pylori* infection in mice, the deletion of gGT has no inhibitory effect on the ability to grow on the culture media [209]. A study using two distinct animal models (mice and piglets) with *H. pylori* infection indicates that in infection with gGT-deficient strains, the colonization is reduced in comparison to the infection with the isogenic wild type strains. The consistent results in these two animal models also suggest that the presence of gGT provides at least some advantage to allow *H. pylori* to infect epithelial cells [210]. The study suggests that *H. pylori* gGT provides a growth advantage within the gut by salvaging extracellular glutathione to obtain cysteine for subsequent protein synthesis and the activity is greatest at a neutral pH [209,210]. Recently, a similar result of significantly higher colonization with gGT-positive strains was suggested by Wustner et al. [211]. Moreover, a recent study described the novel role of H. pylori gGT in autophagy regulation and bacterial internalization in human gastric cells, which suggests the possible role of gGT in the protection of bacteria to commence a persistent infection [212].

*H. pylori* gGT induces apoptosis, inhibits gastric cell proliferation, arrests the cell cycle, and generates reactive oxygen species [76,77,78,213,214] and the significantly higher activity of gGT has been demonstrated in strains isolated from patients with peptic ulcer disease and gastric cancer, which suggests the possible role of gGT in the contribution to severe pathogenicity [214,215]. Furthermore, a recent study revealed the role of gGT in accentuating VacA-dependent vacuolation of epithelial cells [79]. The enhancement of vacuolation by gGT is carried out by the hydrolysis of extracellular glutamine, thereby releasing ammonia which accentuates VacA-dependent vacuolation. This discovery suggests why *H. pylori* with higher activity of gGT exhibits more severe gastro-duodenal diseases.

## 5. Other Virulence Factors Playing a Role in Triggering Pathogenicity

Despite of the above-mentioned well-characterized virulence factors, several studies have depicted the association of other virulence factors in the severity of gastric complications. A recent study conducted by Capitani et al. described the possible role of the HP1454, a secreted protein and component of OMVs, in the Th1 and Th17-mediated inflammatory response in the chronic *H. pylori* infection and associated gastric cancer [216]. In addition to *cag*PAI encoding the T4SS, another putative gene cluster with low G + C content of around 35% encoding the T4SS in *H. pylori* is located within the integrating conjugative elements (ICE). Recently, we reported the role of the specific variants of *H. pylori* ICE T4SS (ICEhptfs) in pathogenicity. In the study, the *cag*PAI-mediated acute inflammation in the antrum and body was ICEhptfs-dependent, and this dependency was also attributed to the higher atrophy score in antrum [217]. The expression of cholesterol glycosyl-transferase (CGT) that depletes cholesterol in infected gastric epithelial cells blocks IFN-gamma signaling and protects bacteria from the inflammatory response. This discovery suggests a novel mechanism of *H. pylori* CGT utilization for the promotion of gastric carcinogenesis [218].

## 6. Whole Genome Sequencing and Genome Wide Association Studies (GWAS) Approach for Virulence Determination

In recent years, the whole genome sequencing (WGS)-based approach from cultured bacterial isolates has emerged as one of the most comprehensive tools for surveillance study, drug resistance determination, and evolutionary analysis of infectious diseases [219,220,221,222,223,224]. This is due to improved sequencing technologies, the user friendly nature, easy availability of reagents, improved accuracy of up to 93%, reasonably lower costs (150EUR per 5 MB genome by Illumina MiSeq), and faster results (nine days versus 21 days) compared to traditional DNA sequencing [221,222,225,226,227]. In addition to these benefits, the WGS also offers a more comprehensive and accurate tool for genome analysis.

Recently, the WGS has been used to characterize a variety of pathogens to determine the novel or existing virulence factors. In a study, Hurley et al. performed WGS-based characterization of 100 *Listeria monocytogenes* strains to determine disease epidemiology, the presence of drug resistance genes, and to determine virulence factors [223]. Similarly, another study was conducted by Edwards et al. to characterize the putative virulence factors in *Plesiomonas shigelloides* [228]. Furthermore, Aly et al. used a WGS-based study to identify genes associated with biofilm formation, antibiotic resistance, and pathogenicity [224]. The results of these studies clearly suggest that WGS-based approaches are an advancement over traditional Sanger sequencing.

Currently, the WGS tool is mostly being used to determine the presence of antibiotic resistance genes in *H. pylori* [229,230,231,232] because of the absence of the resistance determinants in plasmids, transposons, or integrons [233]. However, this approach has recently gained popularity in virulence determination. In a recent study, Ogawa et al. performed WGS of 43 clinical isolates *H. pylori* (17 chronic gastritis, eight gastric ulcers, eight duodenal ulcers, and 10 gastric cancers). Full *cagL* and *cagI* sequences were analyzed for single nucleotide variation and amino acid changes. The WGS results identified several putative novel variants of CagL and CagI sequences, proving its usefulness in virulence determination [234]. The search for the genetic basis of susceptibility to particular diseases using populations with the specific diseases and matched controls and GWAS has become popular in human genetics [235]. Recently, this novel state of the art methodology was used in *H. pylori* to search for the novel virulence factors using genomic information from strains isolated from gastric cancer and matched controls like isolates from the duodenal ulcers. Berthenet et al. used GWAS in their recent study to illustrate the association of the presence of the *cag*PAI genes *cag11* (*cagV*), *cag12* (*cagU*), and *cag 20* (*cagI*) in *H. pylori* isolated from patients with gastric cancer [236].

### H. pylori and Microbiota in Gastric Carcinogenesis

*H. pylori* promotes carcinogenesis by influencing the composition of the microbial population (microbiota) in the stomach [237,238,239]; however, the mechanism is not fully understood. The precise balance of commensal microbiota in the gastrointestinal tract plays a role in the regulation of the host mucosal immune response, efficient killing of potential pathogens, and carcinogenesis [240,241,242,243,244]. Studies have suggested that some of the microbes including *Escherichia coli*, *Lactobacillus* spp., *Nitrospirae* spp., *Clostridium* spp., *Veillonella* spp., *Haemophilus* spp., and *Staphylococcus* spp. that convert nitrogen compounds to potentially carcinogenic N-nitroso compounds in gastric fluid enhance cancer development [238,245,246,247], whereas oral commensals such as *Streptococcus* spp., *Prevotella* spp., and *Neisseria* spp. are associated with a lower risk for gastric cancer development [244,248]. In contrast, other oral microbes such as *Pasteurella stomatis*, *Spodoptera exigua*, *Parvimonas micra*, *Streptococcus anginosus*, and *Dialister pneumosintes* exhibit a strong co-occurrence in gastric carcinogenesis [243]. In a study, Wang et al. observed an abundant presence of five bacterial genera (*Lactobacillus* spp., *Escherichia coli*, *Shigella* spp., *Nitrospirae* spp., *Burkholderia fungorum*, and *Lachnospiraceae* spp.) in gastric cancer patients, where *Nirospirae* spp. was observed in all gastric cancer patients but not in chronic gastritis patients [247]. A recent study has described the alteration in the relative abundances of the dominant phyla Bacteroidetes, Firmicutes, and Proteobacteria in the fecal microbiota of subjects with *H. pylori*-positive gastric precancerous lesions, which suggests the possible association of these phyla with the progression of *H. pylori* related gastric lesions [249]. The co-excluding and co-occurring interactions of *H. pylori* with Methylobacillus and Arthrobacter, respectively, in severe gastritis has been observed, whereas co-excluding interactions with members of the phylum Firmicutes (Ruminococcus, Bacillales, and Lactobacillus) and co-occurrence interactions with Prevotella, Moryella, and another helicobacter (*H. ganmani*) have been reported [243]. Therefore, site-specific gastric microbiota that colonize the tumor microenvironment are an important factor for carcinogenesis and its progression.

## 7. Conclusions

This study depicts the importance of several virulence factors playing a role in the severity of gastric complications. *H. pylori* infections contribute the highest risk for developing severe gastric diseases. Therefore, the virulence factors characterized in the isolated strains will provide a clue for the disease prediction in the populations. Currently, WGS and GWAS-based investigations and their meaningful results for the putative virulence and drug resistance determinants have encouraged researchers to perform more extensive and multidisciplinary efforts for better understanding of virulence factor involvement in the onset and progression of gastric complications.
